# Acute exertional compartment syndrome of the supraspinatus

**DOI:** 10.1308/rcsann.2025.0019

**Published:** 2025-06-17

**Authors:** S Walters, S Yousaf, M Baker, V Patel

**Affiliations:** Epsom and St Helier University Hospitals NHS Trust, UK

**Keywords:** Acute exertional compartment syndrome, AECS, Supraspinatus, Fasciotomy

## Abstract

Acute exertional compartment syndrome (AECS) is increasingly recognised as an emergency presentation requiring urgent surgical intervention. This can theoretically affect any myofascial compartment, but is rare around the shoulder, with very limited literature relating to AECS of the supraspinatus muscle. It is vital for the treating medical team to have an index of suspicion for this condition in patients presenting with acute severe shoulder pain secondary to exertion, and act promptly to assess and investigate. Clinical assessment can be combined with important adjunctive investigations including magnetic resonance imaging and compartment pressure measurement, and if the diagnosis is confirmed or strongly suspected, emergency fasciotomy should be performed. We present a case of a patient with AECS of the supraspinatus, treated with emergency fasciotomy, who made a good long-term recovery.

## Background

Compartment syndrome is the manifestation of increased pressure within a myofascial compartment that leads to local tissue ischaemia and hypoxia, and if left untreated, necrosis giving rise to local and systemic complications.^1,2^ Compartment syndrome can occur as an acute traumatic phenomenon, typically secondary to a fracture or crush injury, or as an exertional phenomenon secondary to repetitive high-intensity muscle contraction, typically in athletes.^3^

This exertional pathology has historically been referred to simply as ‘chronic’ compartment syndrome, but it is now accepted that this can occur in chronic or acute forms.^3^ Chronic exertional compartment syndrome (CECS) is characterised by improvement with rest and no requirement for emergency fasciotomy (although this may be considered electively), whereas acute exertional compartment syndrome (AECS) requires the same urgency in diagnosis and treatment as acute compartment syndrome associated with trauma.^4^

AECS most commonly affects the lower limb, but can occur in any muscle compartment, and its diagnosis and treatment can be challenging in more unusual presentations.^5^ We present a case of AECS of the supraspinatus, which has been described only a handful of times in the literature, and represents an important differential diagnosis for patients with acute severe shoulder pain.

## Case history

A 34-year-old man presented to the emergency department (ED) with a 30-hour history of worsening severe right shoulder pain. He denied any history of trauma, but regularly undertook resistance training and weightlifting, including the day before the presentation. The pain started shortly after this gym session, and became more severe over the following day, prompting his presentation.

He was otherwise fit and well, with no medical comorbidities or regular medications. His ethnicity was Afro-Caribbean, he was right-hand dominant, worked as a builder, was a non-smoker and consumed alcohol only occasionally. He denied the use of any other drugs, including anabolic steroids.

In the ED, he required high doses of intravenous opioid analgesia, and remained in severe pain. Radiographs of his shoulder were obtained to rule out acute calcific tendonitis and were unremarkable. His blood tests revealed a markedly elevated creatine kinase (CK) of 6,667U/L, and only a mildly raised white blood cell count of 13.6×10^9^/L. His C-reactive protein level was 3.5mg/L, troponin I was 7ng/L and renal function was also normal (creatinine 98μmol/L, estimated glomerular filtration rate 86mL/min).

**Figure 1:**
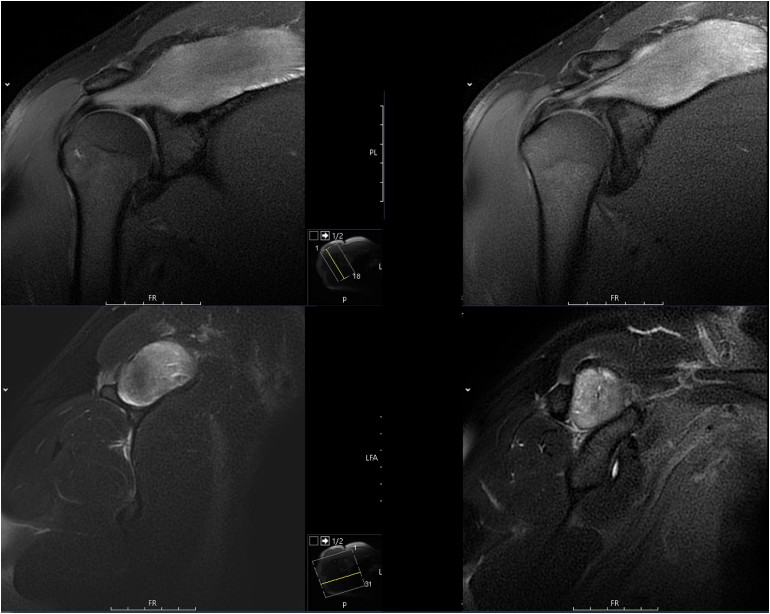
Magnetic resonance imaging of the right shoulder demonstrating increased signal and enlargement of the supraspinatus. Upper, proton density (PD) weighted sequence; lower, T2-weighted sequence.

**Figure 2:**
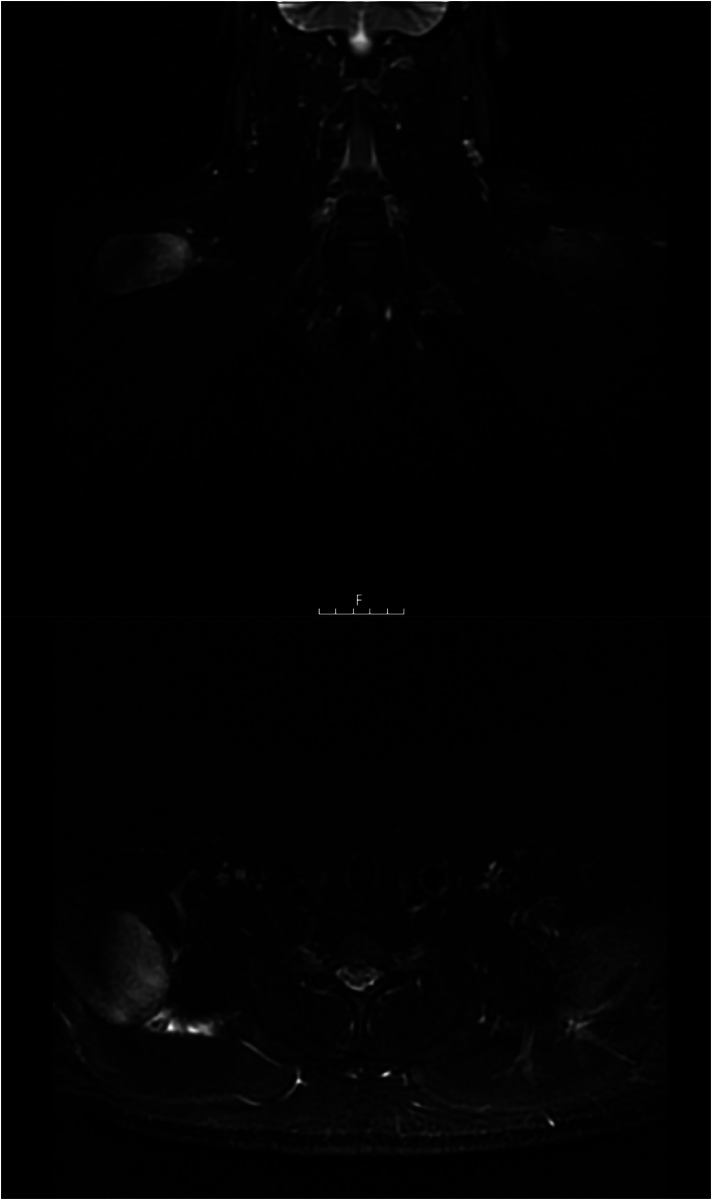
Magnetic resonance imaging of both shoulders demonstrating increased signal and enlargement of the supraspinatus on the right, and mild changes on the left. Short tau inversion recovery (STIR) sequence.

On examination, he had focal swelling and tenderness over the right suprascapular region. He was only able to abduct his arm actively to 45 degrees due to the pain. An urgent magnetic resonance imaging (MRI) scan was organised, which showed bilateral supraspinatus muscle oedema and swelling, more pronounced on the right side (Figures 1 and 2). The suggested differential diagnosis included myositis/autoimmune reaction, or rhabdomyolysis.

Following this, a diagnosis of AECS of the supraspinatus was presumed, and the patient was consented for compartment pressure measurement and surgical intervention. Of note, he denied any significant discomfort on the left side, so intervention was planned for the right side only.

The patient was taken to theatre within a few hours of presentation, and had a general anaesthetic and was positioned in lateral decubitus with the right shoulder exposed. Prior to incisions, compartment pressures were measured, using a Compass UniversalHg device (Centurion Medical Products).^6^ These measurements showed a pressure in the supraspinatus of 38mmHg; the infraspinatus was measured for comparison, and was 4mmHg. The patient’s blood pressure at the time of compartment pressure measurement was 126/55mmHg (after anaesthetic, although similar to preoperative values). Although some advocate for continuous or repeated pressure measurements to assess the trend, this single value (delta P = 17mmHg) alongside the patient’s preoperative symptoms and MRI findings were taken to confirm the diagnosis of compartment syndrome of the supraspinatus, and a fasciotomy was performed.^7^ A limited incision was made over the spine of the scapula (modified Judet approach). The trapezius fibres were split to identify the supraspinatus fascia. Initially, a small incision was made in the supraspinatus fascia, and the underlying muscle was found to be slightly browner and darker in colour than the overlying trapezius muscle. The fascial incision was extended along the muscle to allow full decompression. A biopsy of muscle was taken and sent for histological analysis. No deep sutures were placed, the subcutaneous fat was approximated without tension and the skin was closed with nylon sutures.

Following surgery, the patient’s pain improved. The histology report showed a section of skeletal muscle with some evidence of early necrosis, but no convincing features of myositis. Serial blood tests showed that CK peaked the day after surgery, at 15,192U/L, before decreasing daily to 4,591U/L on day 4 postoperatively. The patient was discharged on day 4 postoperatively, and was seen again in clinic on day 11, at which point his CK was 272U/L.

**Figure 3:**
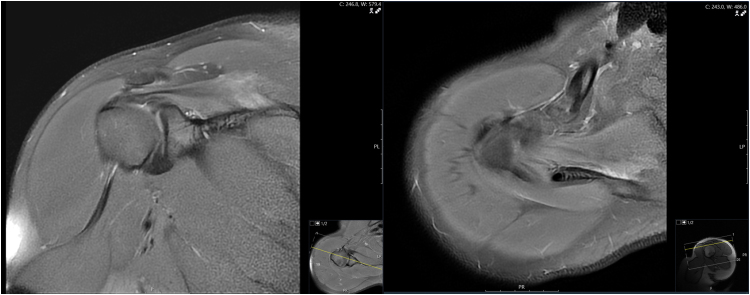
Magnetic resonance imaging of the right shoulder at 4 months post surgery showing reduced signal and size of supraspinatus. Coronal reconstruction (left) and axial (right) proton density (PD) weighted sequence.

The patient had interval MRI scans after 2 and 4 months, which both showed relative improvements in the appearance of supraspinatus, with a reduction in swelling and signal change (Figure 3). At 6 months postoperatively, he was seen in clinic and was almost completely pain-free and had good shoulder function.

At 2-year follow-up, he reported no significant problems with his shoulder. He was back to working unrestricted. He had elected to stop weightlifting, but was planning to start building this back up gradually. He had a full range of movement including full overhead abduction with good power. His Oxford Shoulder Score was 46/48, losing points because he felt an occasional mild pain in the shoulder (Q1), and the shoulder pain was noticeable “a little bit” when using certain tools at work (Q11).

## Discussion

Compartment syndrome affecting the supraspinatus is a rare condition that has limited reports in previous literature. Although it has been reported secondary to trauma (scapular fracture), its occurrence without trauma, but secondary to exertion, was first reported by Takakuwa *et al* in 2000.^8,9^ Another report describes two patients with pathology affecting the supraspinatus.^10^ One of these was treated with urgent surgical decompression once the diagnosis of compartment syndrome was made, based on clinical findings, elevated serum CK and elevated compartment pressure readings. However, the other case was managed non-operatively – this patient had an elevated serum CK level and compartment pressures were not measured, but ultrasonography was performed and reportedly showed “a large haematoma in the supraspinatus but no muscle compartment necrosis”, and on this basis no surgical intervention was undertaken. This patient had some ongoing weakness for several months, but had made a full recovery at the one-year follow-up, both clinically and on an MRI scan. This prompts further comment about the nature of this pathology in the supraspinatus as a spectrum, coined ‘exertional supraspinatus syndrome’, ranging from supraspinatus rhabdomyolysis, which is self-limiting and does not require decompression, through to compartment syndrome, which is characterised by elevated intracompartmental pressure readings and the potential for irreversible muscle and nerve damage if not treated surgically with fasciotomy.

By this definition, there have been a few reports in the literature of supraspinatus rhabdomyolysis that were managed non-operatively with good long-term outcomes.^9–11^ Like our case, there have been others in which a diagnosis of compartment syndrome was made, and emergency fasciotomy was performed, with good long-term outcomes.^12–15^ One reported case is particularly of note, because the patient presented with a similar history of shoulder pain the day after weightlifting, and the MRI scan showed oedema and enlargement of the supraspinatus. This patient underwent emergency fasciotomy, but unfortunately had a poor functional outcome 15 months later, despite extensive physiotherapy, with arm abduction limited to 30 degrees due to pain.^16^ This demonstrates clearly the potential sequelae of compartment syndrome and the need for urgent diagnosis and treatment.

In terms of making a distinction between rhabdomyolysis and compartment syndrome, and therefore deciding whether emergency fasciotomy is required, the consensus in the literature is that compartment pressure monitoring is a key adjunct when it comes to exertional supraspinatus syndrome. This is not necessarily the case for traumatic presentations, in which it should be considered to be primarily a clinical diagnosis, and pressure measurements are used as an adjunct for obtunded patients or those with unreliable examination findings.^1,10,17,18^ In the two cases of supraspinatus rhabdomyolysis presented by Graves *et al* (both managed non-operatively), compartment pressures were not measured for either patient, and the authors justify this because of the delay in presentation from symptom onset (over 24 hours), and the lack of ‘tenseness’ on palpation of the muscle.^11^ We would argue that if there is clinical suspicion of supraspinatus compartment syndrome, compartment pressures should be measured, especially if the intention is non-operative management, because the potential consequences can be devastating.^16,19^

MRI scans have proven value in the diagnosis of exertional compartment syndrome, and given that they are readily available in modern practice, every patient with a potentially compatible presentation should have this investigation urgently.^20^ The majority of previous cases discussed included an MRI scan, and these revealed features of supraspinatus enlargement and oedema, with increased signal intensity on T2-weighted images. However, these abnormalities were also present in the cases considered to be rhabdomyolysis and managed non-operatively.^9,11^ Also of note, in the case we present here, MRI demonstrated abnormalities in the contralateral supraspinatus (Figure 2), but this was asymptomatic. This suggests that radiological changes may be seen in the milder stages of exertional supraspinatus syndrome, when the muscle has not reached sufficient pressure to be symptomatic. It has been shown that changes on MRI are not specific to compartment syndrome, and in CECS, MRI has been reported to be as sensitive as compartment pressure measurement, but less specific.^21^ Therefore although MRI is a useful investigation that helps localise the pathology, it does not necessarily completely direct treatment, and the clinical findings with or without compartment pressure measurements should be used to determine the need for fasciotomy.^2,15^ An MRI scan may also identify alternative pathology, and our case also demonstrates the value of MRI in post-treatment monitoring given that improvement was observed on the follow-up scans (Figure 3).

Emergency surgical decompression with fasciotomy is the universally accepted treatment for compartment syndrome. Hyperbaric oxygen therapy has been suggested as an adjunctive treatment, and was used in one of the aforementioned cases of supraspinatus compartment syndrome.^13^ This remains an interesting area for ongoing research, but currently lacks enough evidence to be routinely incorporated into practice, perhaps also because of its lack of availability in most centres.

In terms of the epidemiology of exertional compartment syndrome, many of the previous reports and literature relate to military patients.^9,11,22,23^ However, the majority of the more recent reports, previously discussed, relate to young males actively involved in weightlifting. With the rise of fitness and bodybuilding culture, we expect that the incidence of this pathology may continue to increase, further stressing the importance of being aware of it and considering it as a potential diagnosis for a patient with an acute onset of severe shoulder pain.

## Conclusions

AECS of the supraspinatus is an uncommon pathology that must be considered in the differential diagnosis for a patient presenting with acute severe shoulder pain and a compatible background and history of exertion. Clinical examination including response to analgesia remains critical, and investigations should consist of plain x-rays, blood tests including CK, and consideration of MRI scanning and compartment pressure measurement if there is any diagnostic doubt. Emergency fasciotomy remains the gold standard of treatment, and when performed without delay, offers the patient the best prospect of making a full functional recovery in the long term.
